# Self-Help Groups within Nursing Homes: The Experiences of Family Caregivers in Northeastern Italy

**DOI:** 10.3390/bs13060485

**Published:** 2023-06-08

**Authors:** Ciro De Vincenzo, Ilenia Marian, Silvia Piol, Shoshi Keisari, Ines Testoni

**Affiliations:** 1 Department of Philosophy, Sociology, Pedagogy and Applied Psychology (FISPPA), University of Padova, 35131 Padova, Italy; ciro.devincenzo@unipd.it (C.D.V.); ileniamarian97@gmail.com (I.M.); silvia.piol.doc@gmail.com (S.P.); 2 School of Creative Arts Therapies, University of Haifa, Haifa 3498838, Israel; skeisari@staff.haifa.ac.il; 3 Emil Sagol Creative Arts Therapies Research Center, University of Haifa, Haifa 3498838, Israel

**Keywords:** older adults, family caregiving, nursing home placement, ambiguous loss, self-help groups, social support, COVID-19, interpretative phenomenological analysis

## Abstract

Older adults and their family caregivers experience nursing home placement as a particularly critical time of life. The present study explored the experiences of family caregivers of nursing home residents taking part in a self-help group for caregivers. The sample was composed of six caregivers of older adults residing in a nursing home in the northeast of Italy. The respondents, aged 57 to 71, were part of a self-help group set up by the facility between 2017 and 2019. In this qualitative methodological design, we applied the principles of interpretative phenomenological analysis. Two main themes emerged from the interviews: (a) challenges in constructing experience as caregivers; and (b) shared experiences as stabilizing tools. The findings highlight the importance of self-help groups in fostering the well-being of caregivers of older adults living in nursing homes. The self-help group enabled caregivers to deal with nursing home placement and the sense of guilt deriving from it; understand and accept the disabilities affecting their loved one; comprehend the experience of ambiguous loss; and learn to listen to their own needs, thus avoiding physical and emotional exhaustion.

## 1. Introduction

Worldwide, contemporary societies are experiencing population aging, i.e., the population distribution is shifting toward older ages [1]. The same is true for the northeast of Italy, where this study was carried out. In Italy, people aged 65 and over make up 23.5% of the population [2]. In the population who are over 80 years of age, 6.3% are nursing home residents [3]. On a regional level, institutionalized care is significantly more prevalent in the northeast of Italy. These data highlight the importance of taking the actors involved in institutionalization, i.e., the older people, but also their informal and formal caregivers into account in policy making. This study was designed to explore the experiences of informal caregivers who placed an older family member in a nursing home.

The difficulties associated with caring for older family members can lead caregivers to develop what is known as the caregiver burden [4]. Caregivers may experience feelings such as sadness, guilt, and uncertainty regarding the future [5,6] that are not only related to difficulties with respect to taking care of the family member, but also to negative emotional reactions when the actions of other relatives fall short of their expectations [7].

In Italy, the social norms of filial obligation toward older parents are strong compared to other Western European countries, and most Italians think that adult children are responsible for providing long-term care support to an older widowed parent [8]. Moreover, Italy has a familial welfare system, in which families are the main providers of care and welfare for dependent individuals [9]. Nonetheless, the way support is provided is rapidly changing. In a study by Albertini and Mantovani [8], almost one third of all participants indicated that hiring a caregiver was the optimal solution to meet their parents’ care needs. Thus, over time, the caregiving of older adults has undergone an inevitable transformation towards institutionalization. This can also be linked to increased life expectancy, the rise in chronic diseases, and the increasing incorporation of women, to whom the exclusive duty of care was once delegated, into the world of work [1,4,10,11]. However, even after nursing home placement, family caregivers still play a significant role in supporting care, and most of them remain very involved in their loved one’s lives [12]. Although there is considerable literature on the experience of caregivers, there is scant literature on the experience of the family caregivers of nursing home residents [13].

While caring for an older adult, caregivers can experience ambiguous loss, which is defined as a loss experienced as unclear and without resolution [14]. Ambiguous loss can occur when a loved one is physically absent but psychologically present (such as in cases of missing persons or kidnappings), or in cases where the loved one is physically present, but their psychological or cognitive abilities are significantly altered, as in Alzheimer’s disease [14]. The present article focuses on the second case. According to Boss [15], the term “loss” is used because a family member is called upon to care for a person whose behaviors he or she no longer recognizes, as a result of the progression of disease. Caregivers may therefore experience anticipatory grief [16]. However, the physical presence of the family member prevents them from dealing with the loss, which often takes place through a language of mourning characterized by rituals and symbols that give meaning to the event, but which cannot be concretized because the person is still physically present [17]. The community is unable to support families going through this experience because it is perceived as destabilizing; namely, society cannot recognize the suffering of the sufferer because it cannot make sense of particular forms of loss. This type of experience can lead to the stigmatization of bereaved families because their grief management practices are not in line with people’s expectations of loss [17].

Caregivers may experience anticipatory grief, not only for the loss of their loved one to illness, but also for nursing home placement [18]. A recent review of the literature suggested that the decision to place an older family member in a facility and the subsequent adjustment period is often accompanied by guilt, depression, feelings of failure, and continuing burden, but also an improvement in quality of life [19]. The sense of guilt experienced by caregivers appears multifaceted and can be linked to person-specific and situational characteristics, such as the level of involvement in care, frequency and quality of visits, and perceptions of the residential long-term care facility [20]. Guilt is particularly pronounced when, prior to placement, caregivers had promised the older adult that they would not place them in a facility [21]. Adaptation to the new situation also depends on whether the caregiver and patient lived together prior to placement [21]. When the caregiver is the patient’s spouse, caregivers find it more difficult to accept the placement [22,23]. A systematic review of interventions to support people with dementia and their caregivers during the transition from home care to nursing home care highlighted some caregivers’ needs that are not covered by available interventions, such as information regarding available care alternatives and financial options, and skills in self-care, such as caring for their own mental and physical health [24]. Thus, more research on the effectiveness of interventions to support older adults and their caregivers during nursing home placement is needed.

Family caregivers can find support in self-help groups for caregivers [4]. According to the American Psychological Association, self-help groups are defined as groups composed of individuals meeting regularly to help one another cope with a life problem [25]. Self-help groups are different from therapy groups in that they are not led by professionals, they do not set a maximum number of participants, and they are free of charge. Unlike groups led by professionals, self-help groups provide unique benefits such as friendship, mutual support, experiential knowledge, identity, and a sense of belonging. Self-help groups allow caregivers to express the pain, discouragement, difficulties related to their loved one’s disease, exhausting care work, and guilt, especially in cases where the older adult has been placed in a nursing home [26,27]. Self-help groups have proved to be of great value in improving the well-being of people under psychological distress as well as of healthcare professionals [28]. To provide quality care to their loved ones, caregivers need to receive support. Studies have shown that caregivers who have access to better social support are healthier and can better care for their loved ones [29,30]. However, less is known about the potential of self-help groups for family caregivers of nursing home residents.

The COVID-19 pandemic made this situation even more complex. The onset of the pandemic in January 2020 had a strong impact on both nursing home residents and their caregivers [31]. According to an article by members of the European Network for Gerontological Social Work [32], forbidding visits by relatives during the COVID-19 pandemic had multiple negative consequences for nursing home residents since these older adults could no longer benefit from the additional care provided by their relatives and friends. The literature points to a worsening of mental health conditions in nursing home residents because of visitor exclusion, such as higher levels of depression and anxiety, but also isolation and loneliness [33]. In addition, especially at the onset of the pandemic, nursing homes were considered dangerous places due to their high mortality rates [34]. In Italy, there was a 43% increase in mortality among nursing home residents in 2020 [3]. The present study explored the experiences of family caregivers of nursing home residents. More broadly, it examined their participation in a self-help group for caregivers.

## 2. Materials and Methods

### 2.1. Qualitative Approach and Research Paradigm

This study used a qualitative methodological design [35] informed by a minimal hermeneutic realist philosophy. A minimal hermeneutic realist stance holds that while the existence of “things” is not dependent on the existence of human beings, the meaning, and nature of reality are determined by the encounter with them [36] Throughout the study, we implemented an interpretative phenomenological analysis (IPA; [37,38]) methodology to explore, understand, and interpret participants’ experiences. IPA is guided by phenomenology, hermeneutics, and idiography.

IPA is phenomenological in that it aims to explore participants’ experience on their own terms [39]. It particularly concerns the participants’ significant experiences. More specifically, IPA focuses on those experiences in which participants feel the urge to make sense of what has happened to them, and thus engage in “hot cognition” (i.e., emotionally loaded forms of cognition).

IPA is interpretative in that it is informed by the theory of interpretation, which is hermeneutic [39]. Starting from an understanding of participants as sense-making beings, the participants’ accounts are seen as the result of their engagement with the sense-making of their experiences. IPA is said to be engaged in a double hermeneutic because the researcher also attempts to make sense of the participants (i.e., second-order sense-making), who is trying to make sense of what happened to them (i.e., first-order sense-making; [28]). The double hermeneutic is expressed through a twofold positionality by the researcher: (a) as an insider, to understand what the experience is like for the participant; and (b) standing alongside the participants, to observe the participants’ experiences from a slightly different angle, in order to gain new insights. The process of interpretation by the researcher unfolds at different, progressively deeper levels [39]. According to IPA, a good interpretation is as close as possible to the terms used by participants during data collection, without resorting (at least in the initial phase) to external conceptual frameworks. Interpretation in IPA is also informed by the hermeneutic circle, which consists of a circular style of thinking describing a dynamic, reciprocal relationship between the part and the whole. Just as to understand a part, one needs to look at the whole, to understand the whole, one needs to look at the different parts that make up the whole [39]. The hermeneutic circle operates at different levels. For instance, to understand a single extract, one needs to read the complete text. IPA is informed by the hermeneutic circle since the different steps of data analysis can be accomplished in a non-linear way, where the interpretation is generated by moving back and forth through the data and their interpretation. Another cornerstone of IPA is the ‘gem’, which Smith [40] defines as “the relatively rare utterance that is especially resonant and offers potent analytical leverage to a study […by providing] strong insight to the experience for the individual and often for the group of participants as a whole”. There are several types of gems: the “shining gem”, which is particularly evident in participants’ words, the “suggestive gem”, requiring more engaged and interpretative work by the researcher, and the “secret gem”, which needs a deeper interpretative effort in order to be highlighted [41].

IPA is idiographic in that it chooses to explore a particular case [39]. IPA aims to understand what the experience was like for this person, and what meaning this person generates about it. For this reason, IPA studies usually only have a small number of participants. IPA studies typically examine homogeneous samples, to explore similarities and differences among participants on a specific detail of the analysis.

### 2.2. Researcher Characteristics and Reflexivity

The research team was made up of five participants: a Researcher of Death Studies and Migration Studies (the first author), a Master’s degree student of Social Work (the second author), a Clinical Psychologist and PhD candidate (the third author), a Researcher of Creative Arts Therapies and Clinical Gerontology (the fourth author), and a Researcher in the fields of Social Psychology, Death Education, and Palliative Care (the fifth author). The second author was doing her curricular internship within the nursing home at the time of data collection. She was in charge of contacting participants. The interviews were carried out by the second author, with the third author as the observer. The first, second, and third authors carried out the data analysis independently. To maintain reflexivity, the authors reviewed and discussed sample quotes during the coding process to ensure that the coding was appropriately applied to the responses. The fourth and the fifth authors supervised the whole process, and, in cases of disagreement, discussed the coding process with the first three authors until an agreement was reached. Throughout the data collection and analysis processes, the research team met regularly to discuss the research process.

### 2.3. Context

This study took place in a nursing home in the Veneto Region, in northeastern Italy, in 2021. The nursing home characteristics corresponded to the study aims, since a self-help group had been set up by the nursing home. The second author was doing her curricular internship in this nursing home. The nursing home had 114 residents. According to their mental health status, behavioral problems, autonomy, and care needs, the residents were divided into two groups. In this nursing home, a self-help group for caregivers of older residents held regular meetings prior to the pandemic. The group was facilitated by a social worker and a psychologist. This group was for both the residents and their family members and was designed to accompany them in the delicate phase of nursing home placement, and in the subsequent phases of the resident’s life in the facility.

### 2.4. Sampling Strategy

This convenience sample was composed of members of the self-help group organized by the nursing home. This group was facilitated by a social worker and a psychologist. The researchers contacted the psychologist and the social worker. Six caregivers agreed to participate.

### 2.5. Ethical Issues Pertaining to Human Subjects

This study adhered to the APA Ethical Principles of Psychologists and Code of Conduct, and the principles of the Declaration of Helsinki. All participants were given a detailed explanation of the objectives of the study and the analysis methodology. They were asked for their permission to record the conversations, to transcribe their answers, and to analyze the content. Participation was voluntary. Participants were guaranteed that the content of their interviews would remain confidential and only those who had provided written signed consent took part. All the names cited below are fictitious (pseudonyms). This study was approved by the Ethics Committee for Experimentation of the University of Padua (7CC15D095CB346DC44FA0F7282C1FE1D).

### 2.6. Data Collection Methods

Data collection and analysis was undertaken from March to May 2021. Data were collected in the nursing home, in an area that ensured privacy and was in compliance with existing regulations for combating and containing the spread of COVID-19. Data were collected in the form of oral interviews. Each interview lasted approximately one hour. A total of six interviews were collected. Since the interviews were conducted during the COVID-19 pandemic, we also included the caregiving experience during that time.

### 2.7. Data Collection Instruments and Technologies

In line with the IPA methodology, a semi-structured oral interview [35] was conducted with the participants. In IPA semi-structured interviews, researchers use an interview schedule, characterized by open questions, to orient the interview. We suggested several topics during the interview to remain aligned with the research goals, but nevertheless, aimed to preserve a less structured format to be able to elicit new topics related to the subjective experiences of each participant [42]. For instance, some of the questions in the interview guide were: (a) “What prompted you to participate in the self-help group organized by the nursing home?”; (b) “How would you describe your experience with your family member’s nursing home placement?”; (c) “What did you think of nursing homes before your family member’s nursing home placement?”; (d) “Regarding your relationship with your family member, how was your relationship before and how has it changed, if at all, after nursing home placement?”; (e) “Do you think that your participation in the group has changed your experience in any way; if yes, how?”; (f) “Do you think COVID-19 has changed your relationship with your family member and how?”.

### 2.8. Data Processing

The interviews with participants were audio recorded and transcribed verbatim. The recordings and the transcripts, together with the informed consents were stored by the principal investigator in a safe place.

### 2.9. Data Analysis

Transcripts of the interviews were analyzed following IPA methodology. In this study, a pencil–paper analysis was carried out, so no software was used. In line with IPA, the data analysis was first carried out case by case and then moved to a cross-case analysis. The case-by-case analysis was structured around six main steps which, can be carried out in a non-linear fashion [39]. During the first and second steps, the researchers immersed themselves in reading and re-reading the text and aimed to create exploratory notes. The latter was an initial exploration of the text carried out by noting anything of interest that produces a set of comments on the data. The researchers created a three-column table, in which they placed the transcript in the middle and the exploratory notes on the right. The emergent themes were placed in the left column. During the third step, the researchers re-read the text together with the exploratory notes and generated emergent themes. The latter were brief concise statements regarding the most important comments linked to a piece of transcript. During the fourth step, the researchers mapped how the themes connected to one another in the experience of a given participant. During the fourth step, they created superordinate themes. During the fifth step, the researchers moved to a different case. During the sixth step, they conducted a cross-case analysis to identify convergences and divergences in the features of the experiences of different participants. In terms of gems (see above), the themes highlighted can be considered suggestive gems.

Finally, the researchers proceeded with the write-up phase, in which, themes that emerged were converted into a narrative account.

### 2.10. Techniques to Enhance Trustworthiness

The researchers created an audit trail (see Supplementary Materials) to show readers how the data analysis was carried out within the IPA methodology. The data analysis was carried out independently by three coders who met regularly to discuss their analyses, and two researchers who supervised and supported the coders in case of divergences.

## 3. Results

Of the six participants, five were female. They ranged in age from 57 to 71 years, with an average age of 64.3 years. Almost all the participants were married with children. One was divorced and one was cohabiting. Four participants were retired and two still worked; their educational qualification in almost all cases was a degree. The participants had been in the self-help group from 2017 to 2019.

### 3.1. Challenges in Constructing Experiences as Caregivers

These challenges related to participants’ difficulties in dealing with dementia and other diseases which prompted them to place their relative in the nursing home. Participants characterized their family member’s illness and the nursing home placement as a disruptive moment, which caused change, discontinuity, and generated conflictual feelings, emotions, and meanings. They struggled to find new, stable modes of experiential organization.

Mara, a retired 71-year-old married woman with two children, placed her mother in a nursing home after her acute onset of Alzheimer’s disease. She described the burden of care that made her opt for nursing home placement: “after all those phone calls, day and night, four–five times a day, I decided that I couldn’t take it anymore”.

Mara’s experience of nursing home placement, similar to that of most participants, was characterized by negative feelings and a sense of guilt:

*“Let’s say, I experienced it (the placement) in a bad way. Even now I am still affected by it. The first period, I cried and she cried, because every time I visited her, it was like a stab because it hurt me to see her in that situation. I was already blaming myself for putting her in the facility”.*

Mara also commented on how little she knew about these facilities: “*I had no idea what a nursing home was like because I had never been in one. The idea of taking mom to this facility was like putting her in ‘prison’”.* She felt the burden of social norms that expect Italian women to take care of their older parents and experienced a sense of self-doubt: she was unsure how to be a good enough daughter:

*“It felt a little bit like parking her there, not being able to take care of her, because in our culture we were taught that you can’t leave them (older adults) in a nursing home”.*

Graziella, a 61-year-old with no children, worked full time. She said she had never been able to accept her mother’s disease, and that she felt anger and a sense of injustice:

*“I’ve never accepted my mother’s disease. It was too big, too big a thing, I said (to the Lord) ‘you could have spared her’. I have this anger inside; it just wasn’t right”.*

When talking about her mother’s nursing home placement, she also described her sense of guilt:

*“It was hard to accept, very hard. After work, I go to visit her, so I feel less guilty, more at peace”.*

Melania, a 63-year-old with one child, was retired and lived with her husband. Melania had physical difficulties taking care of her mother who had Parkinson’s disease: *“If I went on keeping her at home, I would have died”*. This constant care elicited negative feelings including hatred toward her mother:

*“I was constantly there for her. People have to love their parents but cannot completely negate themselves, because then they eventually come to hate them, I felt this way when I had her at home”.*

Melania also described her strong sense of guilt:

*“I started to feel guilty, I kept asking myself ‘did I do (the) right (thing), then she (Melania’s mother) would cry, she would despair... and I kept wondering ‘what should I do? Should I take her home?’”*

When asked about her idea of nursing homes before the placement, she talked about a negative social image of nursing homes and the general lack of knowledge about them:

*“There is hardly any information about what a nursing home is like. It’s still somewhat dictated by the traditional idea that older adults go there to die. They are always portrayed negatively. Stories in the newspaper always describe the bad things. I changed my opinion about them”.*

Giorgio was 71 years old; he was divorced with one son and was retired. He first put his mother in a nursing home because she broke her femur and later because of various physical issues. Giorgio stated he had a hard time with his mother’s nursing home placement:

*“At the beginning mom accepted it well, because she knew she had to stay in bed, but after 5 or 6 years she didn’t want to stay anymore, and it was hard for both of us... she often cried, it was hard... so I would put her in the car and take her for a ride, I was very present... I tried to make her understand that it was just temporary, then we would be together... but it was hard”.*

Laura, a 64-year-old, was retired, married, and had one child. She placed her mother in a nursing home when she was diagnosed with Alzheimer’s disease. Laura experienced negative feelings including anger, confusion, and helplessness when her mother’s disease first emerged:

*“The anger, nervousness, lack of patience because I did not know what was happening. My mother felt sick and asked for help, ‘help me, help me’ she said, ‘Mom how can I help you?’ and I felt helpless. You don’t know who to turn to, how to do it. It feels like you are swimming in a sea, and you are going to drown”.*

Laura went on to describe her anger as follows:

*“I wondered why this was happening to me, “why me? why Mom and Dad”? She (Laura’s mother) was young and could have lived a good life. So, I felt this anger inside”.*

Laura clearly expressed her struggle to accept the role reversal where she had to switch from the one who was cared for, the daughter, to the caregiving one, the mother:

*“Something that was very difficult for me was to take care of my mom. I saw it as unnatural, I could never wash her or feed her because for me a mom is a mom, even if you are 60 years old. It was impossible to accept becoming my mother’s mother”.*

Laura’s feelings about placement ranged from guilt to relief. She said the nursing home placement was a “*hard moment in which you, as daughter, take your mom there, you feel responsible, it feels almost like abandoning her*”. However, she also described a sense of relief from the reduction in her burden of care:

*“It’s like having found a small solution to our big problem. Even though you obviously feel responsible, and your conscience hounds you, at least you can say ‘now there’s someone to help me’; you breathe a sigh of relief”.*

Regina was 57 years old and was the youngest participant. She was married, worked, and had two children. Regina decided to place her mother in the facility because of a disabling disease that led to her paralysis. This caused her significant mental and physical exhaustion, and an embodied burden of care:

*“My mom would have needed 24-hour care. I put her in the facility because I couldn’t take it anymore, I had 3–4 hernias and my mother needed help. We had to take her to the nursing home because I was exhausted. I couldn’t take it anymore”.*

Regina said that “*placing mom in the nursing home gave me real grief*”. She also said that her guilt was linked to betrayal of a promise she made her mother that she would care for her at home:

*“I cried for a year. When my mom retired, she used to say ‘I’m going to a nursing home because my mind’s clear,’ and I used to tell her ‘I’ll never let you go to a nursing home’. Then, I made the decision for her to go. That explains my guilt because I told her she would not go and then I put her there”.*

### 3.2. Shared Experiences as Stabilizing Tools

The participants commented that their sense of disorder and chaos were lessened through their concrete relationship with their family member and with the other members of the self-help group. The self-help group helped the caregivers anchor themselves in constructing their experiences as caregivers.

Mara decided to join the self-help group because:

*“I was so upset about putting my mother in a facility that I needed someone to help me process this transition. I come from a generation where children were expected to look after their parents forever, so I felt so guilty”.*

For Mara, the self-help group enabled her to create meaningful relationships: “*you can make friends*”. The self-help group was an important resource that enabled her to process nursing home placement by sharing her feelings of guilt:

*“When I attended these groups, it helped me a lot to share the pain of separation from home to here (the nursing home). Because we all had the same problem, we all felt guilty, we all had this burden to share. And that’s also what helps us get through it, because if you share the burden, it becomes less of a weight”.*

She also stated that the group made her feel as though they were all “*in the same boat*”, and that there was someone who could fully understand her:

*“Seeing yourself in others... also because people outside tell you ‘I understand’, but no... they don’t understand until they have experienced it. This is sharing things. Others certainly know what the disease is, but no, they haven’t experienced it”.*

She added “*if a psychologist tells you, you don’t believe it, but if a person like you tells you, someone who’s gone through what you’ve been through, it’s different*”.

Graziella stated that the nursing home placement enabled her to increase her physical relationship with her mother.

*“I cuddle more, although still not much. My mom and dad belong to a generation where love was demonstrated through feeding and caring, not through kisses”.*

In terms of her choice and her expectations before joining the self-help group, she said: 

*“Because I knew them and I thought they were a very good way to provide relief to the relatives… Letting them share other people’s pain and understand how the facility works, I think that helps to redistribute the burden of pain, and sometimes we understand there was no other option, since we often feel guilty for placing them in the facility”.*

Graziella described the importance of relationships within the self-help group: “*We were all family at one point and that was already important, having moments of conviviality, it was life*”. She also described the group as an important resource to share her feelings:

*“When you share your own experience, you see yourself in the other person and in a certain way, you can justify feelings such as anger or sadness, and that helped a lot’; that’s what counts”.*

She also added that the group allowed her to realize that other caregivers were going through similar life experiences, which enabled her to benefit from mutual support:

*“When you realize you’re not alone, that many people experience this type of problem, these fears, anguish, when they’re sick and go to hospital... if you share it with the group, it really takes a weight off your shoulders. Also, because we encouraged each other and supported each other psychologically, as much as we could”.*

Melania also talked about the transformation in her relationship with her mother after nursing home placement that allowed them to have a time dedicated to their mother–daughter relationship, as opposed to satisfying caring needs:

*“Since she came here (to the nursing home), she is always kissing me. I was impressed in so many ways by this. I consider it a beautiful thing, because in the last few years there has been a real mother–daughter relationship. I looked after her a lot. We never went out so much as in these three years, having ice cream, etc., so I looked after her, really. There was a time dedicated to her, which was difficult to have at home, but coming here and knowing that there was a time just for us, that made a lot of difference”.*

Melania also added how the COVID-19 pandemic and the social distancing regulations impacted their relationship, and their psychophysical well-being:

*“I went through a crisis because I didn’t see her, I called her, and I think she did too, but then I found her in a bad way... with so many months shut up here, you can only get worse. I found her more absent, older, there was more of a decline in all areas, so much so that three months later she died”.*

Regarding her choice to participate in the group, she said: “*I joined the group because I felt hopeless, since my mother was in a critical situation and felt a lot of guilt”.* She shared she joined the group with the following expectations: “*I wanted to see if it could help, (if being) with people who were already going through the same experience would be helpful, could give me more insights, could be the stimulus to deal with my mother in a different way”.*.

She also reported that the group allowed her to realize that other caregivers had the same feelings and concerns, such as her fear of her mother’s death:

*“I understood I was not the only one to feel remorse, and fear that one day she’ll be gone and then what will be next… We should all understand that one day it will happen, and we all dread this idea”.*

Giorgio also talked about the impact of COVID-19 and the absence of a daily, in-person relationship with his mother that impacted their well-being (*“she would ask me ‘when are you coming here? When are you coming?’, that was hard to bear”*) and his mother’s health:

*“When I saw my mom again after the lockdown, she was distraught, she had lost 10 kg. It didn’t last long after that. COVID-19 had an impact both cognitively and physically. It was a big change”.*

Regarding his choice to join the self-help group, he shared: “*It was something new, I wanted to know what it was about because I’ve always been very present in the facility, I wanted to see if it was something useful... I had gotten a bit of an idea from reading the flyer ... I was driven by curiosity*”. Giorgio described relationships within the self-help group as meaningful:

*“At first, I struggled with sharing in the group, but after years, I didn’t anymore… if you are new, maybe yes, you need to lay bare your fears and it gets complicated, but then you become friends and it goes more smoothly”.*

He also talked about how creating these relationships in the group helped him share his feelings and decrease his sense of burden:

*“You form bonds, and it feels like you can share it (your burden), it’s not just on your shoulders”.*

Laura talked about the transformation in her relationship with her mother after her nursing home placement. As her primary caregiver, she was very nervous; now she could just be with her mother. She appreciated the importance of the time spent with her mother in the nursing home:

*“Something’s changed for me since now I live more calmly; previously, I was always apprehensive, always nervous... now when I come here (to the nursing home), I know that I’m coming to see her, I can be with her that hour* *”.*

Laura also noted the importance of the physical, corporeal, and embodied aspects of the relationship with her mother. Despite her mother’s illness and inability to recognize her, they could still maintain and co-create their relationship through physical contact and care. For this reason, the COVID-19 social distancing regulations had an impact on the relationship between Laura and her mother:

*“My mom has Alzheimer’s, so I can’t communicate with her by talking. I only communicate by touching her, that is, through physical contact. When I first came here after a long time [due to COVID-19], she couldn’t recognize me anymore and I was not allowed to touch her. Then, little by little, I started very cautiously doing her nails, combing her hair, getting into a different relationship... when doing these little things, she looked at me differently. When I came back here the first few times, she would always look for the personnel, but afterwards, she started to recognize me”.*

Laura added:

*“Physical contact is everything, in my opinion, because she hasn’t recognized me as her daughter for a long time, but when I went there, I did her toenails, and she would say to me ‘what would I do if I didn’t have you’. Even if she didn’t recognize me, the affection was there... it was an important channel”.*

Laura decided to join the self-help group, “*In order to get through this very difficult time in which you, as a daughter, take your mother (here). You feel responsible, it almost seems as though you want to abandon her. This is obviously not the case, but you have this sense of anxiety. You feel responsible for this very serious decision*”. Laura considered the self-help group to be a place where she could form meaningful relationships: “*The group is like a family*”. She described the importance of sharing her feelings, where she could feel she was not alone:

*“We all had this anger inside that wears us down. Being able to externalize it helps so much. There were tears, despair, but then it helped so much because the next time you cried less, and you were able to talk more and more. So much so that, after the group sessions, you felt good, relaxed, and discharged. You see so many people going through this; they help you realize that you’re not alone”.*

She added that the self-help group allowed her to tell her story to people she felt could deeply understand her experience:

*“Who do you talk to? People don’t understand. They say they do but they can’t understand unless they go through it. The fact that you can talk to a person who is there (in the nursing home), who experiences this daily, comforts you so much”.*

Regina decided to take part in the self-help group. “*I agreed to participate because we were a good group and also because, for me, placing mom in the nursing home gave me a lot of grief*”. Regina described sharing in self-help groups as a meaningful reference frame:

*“Talking to others helped me a lot, especially for acceptance. We talked to each other; we gave each other advice”.*

She also said that the group was an important way to allow herself to take some free time without feeling guilty:

*“Discussions help you accept the situation. When I started the groups, I had never gone on vacation. This year, they managed to ‘convince’ me to go. They reassured me that mom wouldn’t miss anything and a series of other things... I would never have done that before. They helped me to trust myself and give myself some space”.*

## 4. Discussion

The present study explored the experiences of family caregivers of nursing home residents. In addition, we aimed to understand their experience of participating in a self-help group for caregivers. The main finding points to the potential of self-help groups for caregivers within nursing homes. They can serve as a resource for caregivers during a critical period of transition in their relationship with the family member they are caring for. The data suggest that there is a negative representation of nursing homes, which can be linked to a general lack of awareness and a culture of aging processes in the population.

The participants’ experience with the onset and progression of their relative’s illnesses was a core aspect in the interviews. This was described as a disruptive moment that severely impacted their relationship, which needed to be re-shaped and re-signified. Sometimes, a disease can lead to ambiguous loss, especially in the case of Alzheimer’s disease or dementia, since although the patient is still present, the caregiver must interface with a new person [16,43]. This ambiguous loss, described by Boss [14], was clearly expressed by one participant who noted that “for her, it’s the same if it’s the nurse or me coming”. Ambiguous loss thus impedes the processing of the loss that often takes place through a language of mourning characterized by rituals and symbols that give meaning to the event, but which cannot be realized since the person is still physically present [17].

In the interviews, the caregiver burden emerged as the strongest factor in the decision to place their relatives in the nursing home [6]. This decision often prompted strong feelings of guilt. Studies have shown that when related to anticipatory grief, guilt can be a source of stress for caregivers [44]. As most Italians think adult children should be responsible for providing long-term care to their parents [8], social norms of filial obligation are likely to have impacted the participants’ experience of the nursing home placement and their strong sense of guilt. Guilt is thus a fundamental experience for caregivers, who perceive that they have broken a promise or betrayed the wishes of their family member [21,44]. The difficulty of accepting formal external help out of fear of social stigma appeared to further promote the vicious anger–guilt cycle [44]. All of the participants reported feeling social judgment, as evidenced by their deep distress at not being able to care for their relative. Davis and colleagues [44] suggested that guilt leads to less-well-adapted coping skills and poor adaptation in the individual. However, the interviews also revealed a change in outlook after placement: Many family members acknowledged their choice of nursing home placement as necessary and indeed argued that they would have made the decision earlier if they had known more about how these institutions work. Although experiences of guilt or burden may remain, caregivers may evaluate the placement positively, both for themselves and for the patient [44]. As the interviews also revealed, the transition from primary caregiver to patient advocate and friend can make the visits positive and empowering, and caregivers may come to appreciate the opportunity to spend time with their loved ones in the facility without having to handle their daily caregiving needs [43,45,46].

The interviews highlight the impact of the COVID-19 pandemic on patients’ disease, and their mental and physical health conditions more generally. The literature points to a worsening in mental health conditions (such as higher levels of depression and anxiety, but also isolation and loneliness [33]) and a deterioration in behavioral symptoms due to reduced social contact during the pandemic [47]. A qualitative study exploring how family caregivers of nursing home residents experienced the COVID-19 crisis in nursing homes in Israel found that nursing homes neglected residents’ emotional and social needs, which led to cognitive, physical, and mental deterioration [48]. Another qualitative study found that the closure of social support services led to the faster deterioration of patients with Alzheimer’s, who also became more dependent [49]. Some participants also mentioned how the social distancing regulations meant that physical contact, their preferred mode of communication in Alzheimer’s disease, was curtailed. More generally, the participants noted that COVID-19 impacted the relationship between caregiver and older residents. Italy was hard hit by the pandemic and draconian visiting restrictions were enforced [34]. Most participants mentioned how distraught they were when they could not visit their relatives, similar to reactions reported by Giebel and colleagues [49]. The scientific community has acknowledged the importance of maintaining either virtual or face-to-face relationships between older residents and their family during the COVID-19 pandemic and has criticized the inflexible visiting restrictions enforced by some nursing homes [34,50,51,52].

Difficulties accepting nursing home placement, physical and emotional exhaustion, and the need to share feelings and create meaningful relationships led the participants to seek support from the self-help group. An overview of the influence of self-help groups on family caregivers showed that self-help groups can improve caregivers’ health and psycho-physical wellbeing, and can reinforce their sense of social support [11]. The participants reported some benefits that are specific to these non-professional groups such as friendship, mutual support, a sense of belonging, and experiential knowledge [25,53]. Corlito [54] argued that the group fosters change through a sense of belonging, emotional sharing, and social comparison. As suggested in Yalom and Leszcz’s concept of “universalism” as a therapeutic factor [55], the participants benefited from meeting others who could understand what they were going through and feel less alone. Golden and colleagues [56] noted that these groups are intended to provide caregivers with new ways of observing and approaching reality. Many of the caregivers here acknowledged that the group was the first step toward change with respect to the painful issues related to the disease and caregiving of their family member.

One of the strengths of the present study was that it addressed the potential of self-help groups as a possible intervention for family caregivers of nursing home residents, to support them during nursing home placement and their subsequent adjustment. The main limitation of the present study was the small number of respondents, which did not allow for generalization of the results. In addition, the findings may not be applicable to other cultural contexts. Future research could expand the number of participants and their cultural backgrounds. It would also be useful to compare the effectiveness of self-help groups with other interventions implemented in the same context, with the same goals.

## 5. Conclusions

This study highlights the importance of self-help groups in fostering the well-being of family caregivers of nursing home residents. Groups help caregivers to cope with their family member’s placement in a facility and the complex feelings of guilt that arise as a result, understand and accept the disease from which their loved ones are suffering and the experience of ambiguous loss, and learn to listen to their own needs, thus avoiding physical and emotional exhaustion. The group becomes a place for discussion and relationships, allowing for personal growth and the development of a social support network. Today, there are only a few initiatives to support family members when they place a relative in a residential facility. Often, family members who make this decision are isolated or stigmatized for receiving support, although they have delegated the primary care to others. More information should be made available regarding nursing homes in order to overcome the prejudices and to psychologically support people who find it difficult to deal with their decision.

Multidisciplinary psychological and social interventions should be designed to support patients and family members. The findings of this study show how the suffering of family members can be accepted and processed in the group, thus enabling an improvement in individual wellbeing.

## Data Availability

The data presented in this study are available on request from the corresponding author. The data are not publicly available due to restrictions in relation to privacy.

## Supplementary Materials

The following are available online at https://www.mdpi.com/article/10.3390/bs13060485/s1, Table S1: Analysis of the interviews interpretative phenomenological analysis audit trail.
