# Supplementary material for: Self-Help Groups within Nursing Homes: The Experiences of Family Caregivers in Northeastern Italy
Source: Behav Sci (Basel). 2023 Jun 8;13(6):485. doi: 10.3390/bs13060485 (PMC10295437; doi:10.3390/bs13060485)
Supplement: Supplementary file 1 [file behavsci-13-00485-s001.zip › behavsci-2380372-supplementary.pdf]

ANALYSIS OF THE INTERVIEWS

INTERPRETATIVE PHENOMENOLOGICAL ANALYSIS

AUDIT TRAIL

Participants	Emergent Themes	Super-ordinate Themes	Cross-case Themes
MARA	Home placement as a negative place	Caregivers' experience with respect to nursing home placement	Caregivers' challenge: Reconnecting the dots
	Sense of guilt	Emotional and physical burden of caregiving	
	Physical exhaustion		
	Loneliness		
	Self-doubt	Sociocultural expectations of caregiving	
	Physical connection	Caregiver's positive feelings	The material and collective experience as stabilizing tools
	Relevance of time		
	Sharing with other people	Caregivers' experience with respect to participating in a self-help group	
	Self-help groups		
GRAZIELLA	COVID-19 impact	Caregivers' experience with respect to nursing home placement during COVID-19	Caregivers' challenge: Reconnecting the dots
	Sense of anger	Caregiver's negative feelings	
	Caregiver's physical discomfort		
	Sense of guilt		
	Sense of injustice		
	The time of life	Caregiver's positive feelings	The material and collective experience as stabilizing tools
	Home placement as a positive place		
	Self-help groups	Caregivers' experience with respect to the patient's disease and changes in the relationship<br><br>Caregivers' experience with respect to participating in a self-help group	
	Relational improvement		
	Sharing feelings with others		
MELANIA	Sense of guilt	Caregiver's negative feelings	Caregivers' challenge: Reconnecting the dots
	Caregiver's physical exhaustion		
	Home placement as a negative place		
	Fear of death		
	COVID-19 impact		
	Positive relational transformation	Experiencing transformations in relationships	The material and collective experience as stabilizing tools
	Self-help groups		
GIORGIO	Home placement as an experiential place	Caregiver's negative experiences	Caregivers' challenge: Reconnecting the dots
	Sense of loneliness and home desire		
	COVID-19 impact		
	Death as freedom	Changing in views about death	The material and collective experience as stabilizing tools
	Self-help groups	Caregivers' experience with respect to participating in a self-help group	
LAURA	Caregiver's loneliness	Caregiver's negative feelings	Caregivers' challenge: Reconnecting the dots
	Sense of guilt		
	Anger and affliction		
	Loss of dignity		
	Diagnosis as a turning point in the emotional life		
	Relational transformation	Experiencing transformations in the relationship with the loved one	The material and collective experience as stabilizing tools
	Self-help groups	Emotional legitimation in self-help groups	
	Sharing feelings and emotions within the self-help groups		
REGINA	Physical exhaustion	Negative feelings	Caregivers' challenge: Reconnecting the dots
	Sadness and sorrow		
	COVID-19 impact		
	Sense of guilt		
	Physical contact	Caregiver's needs	
	Home placement as a transitional moment		
	Self-help groups	Sharing as a meaningful reference frame	The material and collective experience as stabilizing tools
